# Three-Dimensional Identification of the Medial Longitudinal Fasciculus in the Human Brain: A Diffusion Tensor Imaging Study

**DOI:** 10.3390/jcm9051340

**Published:** 2020-05-04

**Authors:** Sang Seok Yeo, Sung Ho Jang, Jung Won Kwon, In Hee Cho

**Affiliations:** 1 Department of Physical Therapy, College of Health Sciences, Dankook University, Cheonan 31116, Korea; eangbul@hanmail.net (S.S.Y.); kjwonpt@hanmail.net (J.W.K.); 2 Department of Physical Medicine and Rehabilitation, College of Medicine, Yeungnam University, Daegu 42415, Korea; strokerehab@hanmail.net

**Keywords:** medial longitudinal fasciculus, visual vertical, diffusion tensor imaging, probabilistic diffusion tensor imaging tractography

## Abstract

Background: The medial longitudinal fasciculus (MLF) interacts with eye movement control circuits involved in the adjustment of horizontal, vertical, and torsional eye movements. In this study, we attempted to identify and investigate the anatomical characteristics of the MLF in human brain, using probabilistic diffusion tensor imaging (DTI) tractography. Methods: We recruited 31 normal healthy adults and used a 1.5-T scanner for DTI. To reconstruct MLFs, a seed region of interest (ROI) was placed on the interstitial nucleus of Cajal at the midbrain level. A target ROI was located on the MLF of the medulla in the reticular formation of the medulla. Mean values of fractional anisotropy, mean diffusivity, and tract volumes of MLFs were measured. Results: The component of the MLF originated from the midbrain MLF, descended through the posterior side of the medial lemniscus (ML) and terminated on the MLF of medulla on the posterior side of the ML in the medulla midline. DTI parameters of right and left MLFs were not significantly different. Conclusion: The tract of the MLF in healthy brain was identified by probabilistic DTI tractography. We believe this study will provide basic data and aid future comparative research on lesion or age-induced MLF changes.

## 1. Introduction

The medial longitudinal fasciculus (MLF) is pair of longitudinal bundles of white matter that provide a pathway from brain stem to the cervical spinal cord [1,2,3,4]. The MLF interacts with eye movement control circuits, which are ascending pathway fibers, and some descending pathway fibers within the brainstem tegmentum involved in the adjustment of horizontal, vertical, and torsional eye movements [4,5]. In addition, it is an essential component of conjugate eye movement, including rapid refixation (saccades) and smooth pursuit, as well as vestibulo-ocular reflex and optokinetic reflexes [5]. Three pairs of cranial nerves (CN), that is, CN III—the oculomotor nerve, CN IV—the trochlear nerve—and CN VI–the abducens nerve—interconnect with the MFL to control coordinated and synchronized eye movements [1,3,4]. The MFL is also known to have connectivity with the medial vestibular nucleus and adjacent ventral lateral vestibular nucleus, which are both related to vestibulo-ocular reflex (VOR)-associated head movements [4,5]. 

MLF lesions can cause neurological impairments, such as internuclear ophthalmoplegia (INO), nystagmus, abnormal vertical eye movements, and ocular tilt reaction impairment [6,7,8,9]. INO is one of the most common signs of an MLF lesion, and manifests as slow or absent ipsilateral eye adduction, with involuntary jerky eye movements (nystagmus) of the abducted eye [4,6,10,11]. Many pathologic studies conducted using neuroimaging techniques have addressed the relationships between MLF lesions and neurological impairments like INO [2,4,12,13,14]. Recently, diffusion tensor tractography (DTT), a technique derived from diffusion tensor imaging (DTI), has been demonstrated to provide the visualization of neural pathway through the diffusivity of water molecules and investigate the white matter integrity [15,16,17]. Therefore, some researchers have used the DTI to evaluate the injuries of brain regions and plan the intervention strategies [12,15,16,17,18,19]. In addition, DTI has been used to visualize and localize the MLF at the brainstem level in three dimensions [2,4]. Diagnoses of MLF lesions using DTT have the advantage of enabling the determination of the aspect of MLF injury more precisely. However, previous studies reconstructed the MLF using the single-tensor tractography, and there was no study that reconstructed the MLF of normal healthy adults using the multi-fiber tractography [2,4]. In 2019, Xie et al. compared the trigeminal nerve tractography according to type of tensor tractography. As a result, they reported that the multi-fiber tractography could provide more sensitive tracking performances and identify the smaller regions and tracts [20]. 

Therefore, in the current study, we attempted to identify and investigate the anatomical characteristics of the MLF in the human brain, using single-fiber and multi-fiber tractography.

## 2. Methods

### 2.1. Subjects

Thirty-one normal healthy adults (aged 20–40 years) with no history of a neurological or musculoskeletal disease were recruited for this study (Table 1). Inclusion criteria were as follows: (1) no medical problem history associated with vestibular function; (2) no history of musculoskeletal, neurological, or cognitive dysfunction; and (3) participants that there were no symptoms related to INO and (multiple sclerosis) MS, and normally reacted to the VOR-associated head movement by evaluating the eye tracking using the LooxidVR (Looxid Labs, Seoul, South Korea). Exclusion criteria for this study were as follows: (1) participants who had previously been diagnosed with the musculoskeletal and neurologic problems; (2) participants who were diagnosed with problems related to brain injury by doctors. All participants provided informed consent prior to DTI. The study was approved beforehand by the institutional review board of Yeungnam University Hospital.

### 2.2. Diffusion Tensor Image Tractography

DTI data were acquired using a 6-channel head coil on a 1.5 T Philips Gyro scan Intera unit (Philips, Best, The Netherlands) with single-shot echo-planar imaging. For each of the 32 non-collinear diffusion sensitizing gradients, 67 contiguous slices parallel to the anterior commissure-posterior commissure line were collected. The imaging parameters used were as follows: acquisition matrix = 96 × 96; reconstructed matrix = 192 × 192; field of view = 240 × 240 mm^2^; TR = 10,726 ms; TE = 76 ms; parallel imaging reduction factor (SENSE factor) = 2; EPI factor = 49; b=1000 s/mm^2^; NEX = 1; and a slice thickness 2.5 mm with no gap (acquired voxel size 1.3 × 1.3 × 2.5 mm^3^) [21].

### 2.3. Fiber Tracking

**Figure 1:**
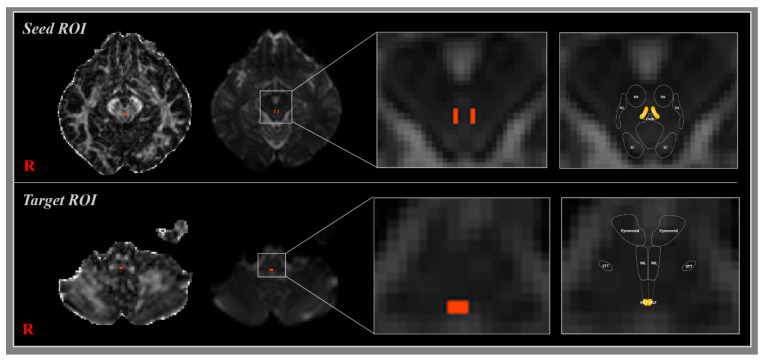
Seed region of interest (ROI) was placed in the interstitial nucleus of Cajal at the level of the midbrain. The target ROI was place on the medial longitudinal fasciculus (MLF) at the level of the medulla (orange rectangle).

Two different methods of DTI were used for reconstruction of the MLF: single-fiber tractography and multi-fiber tractography. In multi-fiber tractography, the Oxford Centre for Functional Magnetic Resonance Imaging of the Brain (FMRIB) Software Library (FSL: www.fmrib.ox.ac.uk/fsl) was used to analyze diffusion-weighted imaging data. The head motion effect and image distortions due to eddy currents were corrected by affine multi-scale two-dimensional registration. Fiber tracking was performed using a probabilistic image method based on a multi-fiber model, and by utilizing image routines implemented in FMRIB Diffusion (5000 streamline samples, 0.5 mm step length, curvature threshold = 0.2) [22]. In the single-fiber tractography, we evaluated the MLF using DTI-Studio software (CMRM, Johns Hopkins Medical Institute, Baltimore, MD, USA), developed for single-fiber tractography (FA < 0.2, angle change > 60°). MLFs were determined by selecting fibers passing through a single seed region of interest (ROI) and a target ROI. Seed and target ROIs were located as follows: a seed ROI was placed on the interstitial nucleus of Cajal at the midbrain level [23,24], and a target ROI on the MLF at the medulla level (Figure 1) [1,25]. A total of 5000 samples were generated from a seed voxel, and the results were visualized at a minimum of 1 streamline through each voxel. Factional anisotropies (FA), mean diffusivities (MD), and tract volumes (voxel number) of MLF were acquired. In addition, we calculated proportion of reconstructed MLF to the target ROI at the level of medulla—the number of voxels analyzed in the target ROI.

### 2.4. Statistical Analysis

The independent t-test was used to determine the significant differences between the DTI parameters of reconstructed right and left MLFs. The Pearson correlation test was used to determine the correlation between age and DTI parameters. The analysis was conducted using SPSS Ver. 20.0, (SPSS, Chicago, IL, USA), and the statistical significance level was set at *p* values < 0.05.

## 3. Results

### 3.1. Multi-Fiber Tractography

**Figure 2:**
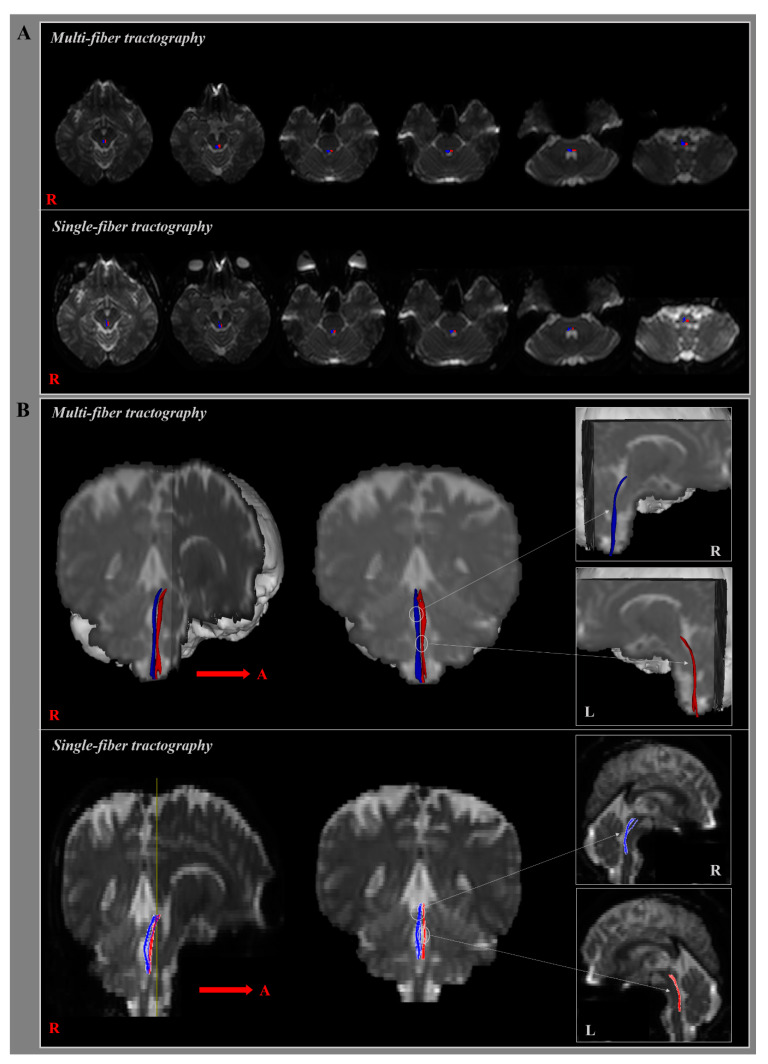
(**A**) The MLF resulting for B0 maps is shown at each level of the brainstem in a healthy subject according to type of DTI tractography. (**B**) The right (blue) and left MLF (red) are shown in coronal and sagittal plane in a healthy subject, using single and multi-fiber tractography.

**Figure 3:**
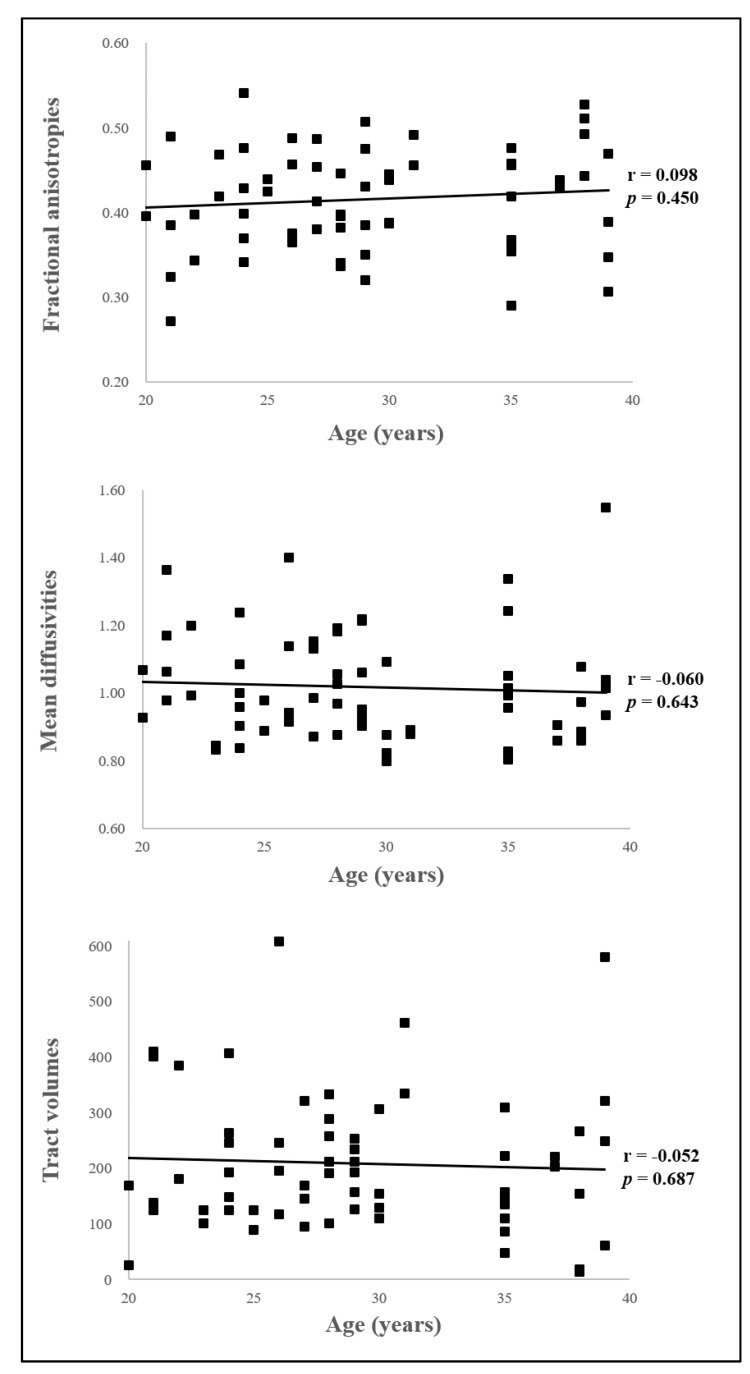
Correlation between age and diffusion tensor imaging (DTI) parameters**.** There was no significant correlation between age and all DTI parameters (*p* > 0.05).

In the present study, we reconstructed MLFs in healthy human brains using multi-fiber tractography (Table 2) (Figure 2). The component of the MLF originating from the midbrain MLF descended through the posterior side of the medial lemniscus (ML) close to CN Ⅵ in pons, and terminated on the MLF at the medulla level at the posterior side of the ML at medulla midline in all 31 subjects (Figure 2A). At the thresholds of 1 streamline, the reconstructed MLF showed connection of 82.97% to the target ROI at the level of medulla. In addition, the reconstruction rate of MLF using the multi-fiber tractography reported 100%. Mean FA, MD, and tract volume values of right MLFs were 0.41 ± 0.06, 1.03 ± 0.15, and 193.84 ± 113.22, respectively, and the corresponding left MLF values were 0.42 ± 0.06, 1.01 ± 0.17, and 223.74 ± 133.74, respectively (Figure 2B). FA, MD, and tract volumes of right and left MLFs were not significantly different (*p* > 0.05). In addition, there were no significant correlation between age and all DTI parameters (*p* > 0.05) (Figure 3).

### 3.2. Single-Fiber Tractography

In the current study, we reconstructed MLFs in healthy human brains using single-fiber tractography (Table 2) (Figure 2). Although the component of the MLF was identical to the result of the multi-fiber tractography, the reconstructed MLF reported the connection of 46.57% to the target ROI at the level of medulla (Figure 2A). In addition, the reconstruction rate of MLF using the single-fiber tractography reported 67.74%. Mean FA, MD, and voxel number of right MLFs were 0.50 ± 0.05, 0.81 ± 0.08, and 69.39 ± 36.59, respectively, and left MLF values were 0.50 ± 0.05, 0.79 ± 0.07, and 67.35 ± 41.18, respectively (Figure 2B). FA, MD and voxel numbers of right and left MLFs did not show significant differences (*p* > 0.05). 

## 4. Discussion

In the present study, we investigated reconstructions and images of MLFs in healthy human brains, using single and multi-fiber tractography. The MLF contains fibers related to the control of the head position and visual vertical project that pass through the MLF to the spinal cord [1,25,26]. Although the MLF consists of various regions of brainstem, projections related to visual vertical in the human brain terminate in regions of MLF at the midbrain, pons, and medulla levels in brainstem [13,23,27,28]. We selected two ROIs for MLF reconstruction, that is, a seed ROI located on the interstitial nucleus of Cajal at the midbrain level [23,25], and a target ROI on the MLF at the medulla level [1,25]. Reconstruction showed fibers descended from the MLF in midbrain to spinal cord as terminating the MLF at the level of the pons and medulla. Unfortunately, few DTI studies have reconstructed fiber passing through MLFs in healthy human brains, which prevented our comparing MLF anatomies and DTI parameters.

Nevertheless, several neuroimaging and tractography studies have visualized and defined the MLF regions [1,29,30,31]. Zwergal et al. reviewed the anatomy of the parallel vestibular pathway in monkeys, and concluded the MLF might carry vestibular information to midbrain and vestibular-perceptive information to thalamus. In addition, they visualized the MLF at the medulla and pons level [31]. In 2014, Sakai et al. summarized the basic anatomy of brainstem, with the exception of diencephalon, and delineated the region related to control of eye movement using T2 weighted images and a DTI derived vector color map. They reported that CN III, IV, and VI were involved in eye movements and passed through the MLF, and determined that the MLF was located to brainstem at midbrain, pons, and medulla [1]. Their exact locations were as follows: the MLF was located close to midbrain midline and adjacent to CN III nuclei at the midbrain level; located on the posterior side of the pontocerebellar tract close to the ML in the pons; and located at the posterior side of ML at the medulla midline [1,25]. However, these studies did not reconstruct the neural pathway passing through the MLF region. Although many previous studies suggested that the exact location of MLF in the MRI and aspect of injury of MLF in the patients with MS [3,8,32,33], however, that it is difficult to identify injury of MLF with only MRI in patients with stroke or traumatic brain injury [34,35,36].

Previous studies have used DTI to describe the functions and anatomy of the MLF in human brains in the presence of pathologies [2,4,12,13,14]. In 2008, Fox et al. identified the MLF in patients with MS using DTI (without a vector color map) and compared the DTI parameters of MS patients and healthy controls [14]. They observed MD values were elevated in patients, and that MLF in midbrain were correlated with function [14]. In 2015, McNulty et al. investigated fiber tractography images of the MLF in MS, with respect to the impact of INO using a single fiber DTI model [4], and suggested MLF lesions were localized to pontine and midbrain, and recommended future research be conducted using a multiple fiber DTI model [4]. Sakaie et al. [2016] compared DTI-derived MLF fiber tractography findings of MS patients with chronic INO and healthy controls [2], and concluded some patients had MLF lesions in the medulla-pons region, and others had lesions in midbrain. They concluded that DTI could detect small MLF pathway lesions in patients with INO [2]. Although previous studies have described MLF anatomy in patients, especially in MS patients, few multi-fiber tractography or DTI vector color map studies have been conducted on healthy adults. 

In summary, based on single and multi-fiber tractography findings, we identified the tract of the medial longitudinal fasciculus in the healthy human brain. We believe that our results will provide basic data that be found useful in future studies on lesion- or age-induced MLF changes. In addition, we suggested that the diagnosis of MLF injury using multi-fiber tractography would be useful in patients with brain injury, compared with single-fiber tractography. However, the present study has several limitations. First, the generalization of our results is difficult, because of the limited age range of the subjects recruited (20–40 years), and thus, additional research needs to be conducted on a population with a greater age range to produce standard data. Second, the fiber tracking technique is operator dependent. Third, it was difficult to locate ROIs accurately, because of the diminutive sizes of the MLF areas in the medulla and midbrain. Although DTI is a powerful anatomical imaging technique that enables gross fiber to reconstruct, it does not image all fibers. Therefore, we suggest additional studies be undertaken to demonstrate the reliability and validity of MLF, and of its clinical correlations using multi-fiber tractography. In the further research, it is necessary to conduct the study reconstructing the MLF of INO and MS subjects. 

## Figures and Tables

**Table 1:** Demographic data of the normal healthy adults.

	Normal Healthy Subjects (*n* = 31)
Age (years)	29.13 (5.74)
Gender (Male/Female)	17/14
Education (years)	16.35 (1.05)
MMSE (score)	29.94 (0.36)

Values represent mean (± standard deviation).

**Table 2:** Diffusion tensor imaging (DTI) parameters of medial longitudinal fasciculus according to type of DTI tractography.

	Multi-Fiber Tractography	Single-Fiber Tractography
FA	0.42 (0.06)	0.50 (0.05)
MD	1.02 (0.16)	0.80 (0.08)
Tract volume (voxel number)	208.79 (123.81)	68.37 (38.65)

Values represent mean (± standard deviation). FA, fractional anisotropy; MD, mean diffusivity.
